# Development and initial validation of the perceived instrumental effects of violence in sport scale

**DOI:** 10.3389/fspor.2024.1355958

**Published:** 2024-02-27

**Authors:** Sylvie Parent, Stephanie Radziszewski, Allyson Gillard, Ariane Bélanger-Gravel, Marie-Hélène Gagné, Elisabeth St-Pierre, Tine Vertommen, Andrea Woodburn

**Affiliations:** ^1^ Research Chair in Security and Integrity in Sport, Université Laval, Québec, QC, Canada; ^2^ Department of Physical Education, Faculty of Education, Université Laval, Québec, QC, Canada; ^3^ International Research Network on Violence and Integrity in Sport (IRNOVIS), Antwerp, Belgium; ^4^ Interdisciplinary Research Center on Intimate Relationship Problems and Sexual Abuse (CRIPCAS), Montréal, QC, Canada; ^5^ Équipe Violence Sexuelle et Santé (ÉVISSA), Université du Québec à Montréal, Montréal, QC, Canada; ^6^ Department of Information and Communication, Université Laval, Québec, QC, Canada; ^7^ School of Psychology, Université Laval, Québec, QC, Canada; ^8^ Safe Sport Lab, Center of Expertise Care and Well-Being, Thomas More University of Applied Sciences, Antwerp, Belgium; ^9^ Department of Physical Education, Ghent University, Ghent, Belgium

**Keywords:** validation, violence, sport, beliefs, maltreatment, scale

## Abstract

**Introduction:**

A growing body of research is looking into risk factors for interpersonal violence (IV) in sport. This research suggests the existence of several important risk factors, especially organizational and social factors. One of these factors is the beliefs regarding instrumental effects of violence. Coaches may want to drive performance, deter failure, test resilience and commitment, develop toughness, assure interpersonal control, and promote internal competition. In sum, available evidence suggests the risk of IV increases when coaches believe in the effectiveness of strategies involving IV to enhance athlete performance or perceive external approval for these practices.

**Methods:**

The studies presented in this article seeks to develop and validate the Perceived Instrumental Effects of Violence in Sport (PIEVS) Scale in order to measure those beliefs in coaches. In study 1, item generation, expert consultation, cognitive interviews, pilot test and item reduction phases led to 25 items for the PIEVS around six dimensions. In study 2, exploratory factor analysis (EFA) was conducted with 690 coaches in order to determine the PIEVS factorial structure and the convergent and divergent validity of the scale was tested (long and short form).

**Results:**

Our results suggested a one-factor solution for the PIEVS (25 items). This one-factor model provided an excellent fit to the data and a very good internal consistency. The PIEVS and empowering motivational climate were negatively correlated, which supported divergent validity as expected. The PIEVS was positively correlated with the disempowering motivational climate and with sport ethic norms, which supported convergent validity as expected.

**Discussion:**

These findings provide preliminary evidence for the appropriateness of the PIEVS Scale to measure perceived instrumental effects of violence in coaches.

## Introduction

In the context of sport, recent prevalence studies clearly show that interpersonal violence (IV) is a problem, varying between 44% and 86% [e.g., (1–4)]. IV involves the intentional use of physical force or power against other persons by an individual or small group of individuals (5). While IV in this context can be perpetrated by people known (e.g., peer athletes, coaches, parents, entourage members) and unknown to the athletes (e.g., spectators), special attention to IV by authority figures in sport is warranted, especially because of their position of power and trust relative to athletes. Previous studies on interpersonal violence (IV) toward athletes participating in organized sport clearly show high prevalence estimates for all forms of IV from authority figures in sport (1–4, 6–8).

Although IV by authority figures in sport can manifest itself through acts also observed outside of sport (e.g., sexual abuse, physical abuse), some manifestations are specific to the context of organized sport. For instance, there are instrumental forms of IV used by coaches, where actions are taken (e.g., forcing an athlete to use performance-enhancing drugs) or neglected (e.g., denying access to appropriate medical care in the event of an injury) to enhance sport performance or ensure discipline (9, 10). Such manifestations have been referred to as instrumental violence (10), which “consist of both psychological and physical behaviors displayed by a coach that seem to be performance-related” (p. 401). Even though these types of behaviors can be perceived as useful or necessary in sport, they are potentially harmful (11, 12) and negatively related to athletes' motivation (13) and thus problematic.

In a systematic literature review, Roberts et al. (14) identified four important social factors associated to IV in sport: conformity to dominant values, organizational tolerance, organizational stressors, and perceived instrumental effects of violence. This last factor refers to the belief that violence “is functional for motivating athletes and making them perform better” (p. 11) and is composed of eight dimensions. Six dimensions are related to goals intended by authority figures when they use violence, intentionally or not (see Table 1). Coaches may want to drive performance, deter failure, test resilience and commitment, develop toughness, assure interpersonal control, and promote internal competition. The other two dimensions are related to actions of athletes that may want to promote team cohesion by using hazing (abusive team initiation) and impair competitor performance to enhance their chances of winning.

**Table 1:** Definitions of the perceived Instrumental effects of violence dimensions.

Dimension	Definition
Drive performance	Motivates athletes, increasing their efforts and thus improving their performance.
Deter failure	Creates a fear of failure that reduces poor performance.
Test resilience and commitment	Promotes adaptability and tests athletes’ dedication.
Develop toughness	Increases psychological and physical endurance.
Interpersonal control	Reinforces discipline and promotes respect through fear.
Promote internal competition	Increases competition between athletes on the same team or club.

Adapted from Roberts et al. (14).

While the terminology of instrumental violence is relatively new, examples of its use within sport is widespread in the literature. For example, Willson and Kerr (12) studied the concept of body shaming as a form of abuse toward athletes. Their results show that athletes experienced negative verbal comments about their bodies, body monitoring (i.e., regular weigh-ins and constant observations), forced restriction of food and water, public criticism of their bodies, and punishment when body-related standards were not met. In 2014, Pinheiro and colleagues documented underlying reasons for the use of abuse toward young gymnasts. They discovered that some coaches verbally abuse athletes to control their weight, force them to train despite injury and pain, and even resort to the use of corporal punishment of athletes when athletes are not successful in exercises or routines. Another manifestation of instrumental violence is the use of exercise as a form of punishment. Kerr et al. (15) showed in their study that coaches think that this strategy will be beneficial for a multitude of reasons, namely learning, deterrence, motivation, team cohesion, increasing conformity within the team, and increasing well-being, mental toughness, and resilience of athletes. These behaviors from coaches are often rooted in their own athlete experience (16). Developing and strengthening mental toughness, as mentioned earlier, is often a justification for the use of emotional abuse. In their study, Owusu-Sekyere and Gervis (17) have documented many examples of methods described by coaches for developing this “quality” in athletes, namely exposure to emotionally and physically challenging situations such as bullying, belittling, humiliating, shouting, ignoring/isolating, and intimidating. As a last example, McDonald & Kawai (18) reported that punitive techniques such as slapping, punching, hitting with equipment, beating with sticks, or running without water or breaks are used in the context of Japanese sport and serve as strategies to correct errors and improve technique. Globally, those examples of IV could be considered as instrumental violence because of the performance-related goals they seem to serve.

Interestingly, many authors reported a deep-rooted normalization of these behaviors in coaches, as well as in athletes and parents (15, 17–20). Such behaviors are not only normalized, but also sometimes even glorified, and considered as necessary for performance outcomes or to prevent unwanted behaviors from athletes (12, 18, 19). It is also very important to note that abusive behaviors are often perceived as having a positive impact on performance in conditions where athletes are already excelling. Coaches (but also athletes and other sport actors) might interpret this relation as causal; abusive behaviors seem to lead to good performance, which in turn justifies the use of these behaviors (21). Stirling and Kerr (11) explained that the positive or negative perception of abuse seems to be based on the athletes' results: if they perform, such behaviors are seen as acceptable, beneficial, or desired; if athletes underperform, less so. Performance can be used to justify, legitimize, or compensate for emotional abuse.

The normalization of IV behaviors by coaches occurs in an interpersonal and social context specific to sport. Some aspects of this context could influence a coach's beliefs on the instrumental effects of IV toward athlete. Indeed, social and relational factors such as the sport ethic norms (22, 23) and the coach-created motivational climate (24) have been associated with IV in sport. In some qualitative studies, authors also pointed out that IV used by coaches could be influenced by sport ethic norms (18, 20, 25). Coakley & Donnelly (26) defined the sport ethic as “a set of norms accepted as the dominant criteria for defining what it means, in their social worlds, to be defined and accepted as an athlete in power and performance sports” (p. 155). In terms of coach-created motivational climate, Ohlert and colleagues (24) demonstrated that a disempowering climate could constitute a risk factor for IV and, conversely, an empowering climate could serve as a protective factor. Coaches who create an empowering climate focus on the autonomy and social support of the athlete while coaches who create a disempowering climate focus on controlling behaviors (24). Aspects of control are also present in IV behaviors, which are all related to coaches' goal of achieving optimal performance.

In sum, available evidence suggests that the risk of IV increases when coaches believe in the effectiveness of strategies involving IV to enhance athlete performance or perceive external approval for these practices (27, 28). Taking this into account, we assume that beliefs and attitudes could be associated with actual IV behaviors toward athletes, yet were unable to find any study that clearly showed this association. As stated by Roberts et al. (14), there are very rich descriptions of these practices in qualitative research. However, “longitudinal, quantitative research needs to be conducted to cross-validate the current findings” (p. 17). For that purpose, a tool that measures perceived instrumental effects of violence in sport is needed. The present study seeks to develop and validate a measurement instrument that assesses coach beliefs regarding the instrumental effects of violence.

## Material and methods

The *Perceived Instrumental Effects of Violence in Sport* (PIEVS) Scale was developed to measure coaches' beliefs concerning the instrumental effects of various IV behaviors. The development and initial validation of the PIEVS occurred in two complementary studies following DeVellis (29) guidelines for scale development. Ethical approval from the principal investigator's university was obtained for each study presented below (blinded for review).

### Study 1: Scale development

#### Item generation

An initial pool of items was developed based on a recent systematic literature review which included the concept of perceived instrumental effects of IV, defined by Roberts et al. (14) as the belief that violence “is functional for motivating athletes and making them perform better” (p. 11). Based on the work of Roberts et al. (14), we generated items based on the six dimensions presented earlier in the introduction, namely *drive performance*, *deter failure*, *test resilience and commitment*, *develop toughness*, *assure interpersonal control* and *promote internal competition*. Roberts et al.'s definitions for each of the six dimensions are presented in Table 1. Following an iterative process, the items were developed from these definitions by two research assistants and the principal investigator. This led to an initial pool of 87 items, in French, that were divided among the dimensions (goals). In terms of response format, a five-point Likert scale was designed, ranging from: (1) Strongly disagree, (2) Disagree, (3) Neither agree nor disagree, (4) Agree, to (5) Strongly agree.

#### Item justification

##### Expert panel

The initial pool of 87 items was revised by a panel of six researchers with various expertise relevant to the study (e.g., coach development and maltreatment inside and outside of sport), to improve construct validity (29, 30). Three of the experts were part of the research team [initials blinded for review] and three others were independent from the study. They had between 5 and 23 years of experience as professors with complementary proficiency related to beliefs about the instrumental effects of IV in sport and psychometric questionnaire development. Each expert was asked to assess the items regarding three distinct criteria on a scale of 1–4 and had the opportunity to provide comments. They scored relevance, meaning to what degree the items referred to the concept of belief in instrumental effects of IV, for which a definition was provided. They also scored clarity, meaning the degree to which the item is easy to understand, and the vocabulary is adapted to the coaches who will answer the scale. Finally, they rated conciseness, meaning to what degree the item is of optimal length or could be shortened. Based on their input, 22 items deemed less relevant or redundant were removed. For example “Insulting or humiliating an athlete is a good test of their level of devotion to their coach” was considered confusing because of the use of the word test. One expert also questioned if the devotion was toward the coach or toward the sport. Another 21 items were modified due to grammatical or comprehension issues. For most, experts suggested reformulations to be more concise or add precision. Finally, two items were added to include specific aspects that were not covered. A total of 65 items remained across six dimensions in the PIEVS.

##### Cognitive interviews

Cognitive interviews were then conducted to assess the scale response process (30–32). The goal was to assess the validity of each item, based on the participants’ cognitive processes when reading the item, understanding the item, evaluating their answer, and formulating the answer (31, 32). The interviews were conducted with five purposively sampled coaches who read the items aloud and shared their reflections with a research assistant. The coaches’ answers were then analyzed using an item-based analysis inspired by Peterson et al. (33). Two research assistants and the principal investigator discussed adjustments to be made to increase item's clarity. A total of 12 items were modified for clarity and three were removed, resulting in 62 remaining items. For example, one item concerning yelling as acceptable to motivate athletes and participants wondered if yelling encouragements would be included. To add precision, the item was changed to “yelling or swearing” to indicate the negative undertone.

##### Pilot test

A pilot of the 62-item PIEVS was then conducted to further identify the most relevant items as well as to evaluate the completion time. The goal was to reach a more concise version and to identify potential issues before the validation study. We purposively sampled 29 participants from four university sport programs and one community sport organization. This choice was made to preserve the larger coach sample for the final validation study. Indeed, the PIEVS was included in a larger questionnaire to test the completion time for an upcoming study of which the validation was one of the objectives. Informed consent was obtained for each participant. The questionnaire was administered online, using Qualtrics software. Most participants were male (*n *= 19) and aged between 26 and 35 years old (*n *= 13). They were active in 11 different sports, the most frequent being badminton (*n *= 7), soccer (*n *= 7), and gymnastics (*n* = 5). The majority coached either at a provincial (*n *= 12) or local/regional (*n *= 11) level. Descriptive statistics were performed for each item.

#### Final item reduction

Given the low number of participants for the pilot, we were unable to proceed to preliminary psychometric testing of the items (e.g., inter-item and item-total correlations). Choices were therefore made based on the whole process, going back to the literature used to develop the initial pool of items, the experts’ and participants from the cognitive interviews comments as well as the descriptive analysis of the pilot. The principal investigator gathered the results from the previous steps to make the final choice of items for the PIEVS. Given the length of the scale and the long completion time during the pilot test, 37 items were removed based on relevance or redundancy. As a result of the development study, the PIEVS consisted of 25 items across six dimensions.

### Study 2: Initial scale validation

An exploratory factor analysis (EFA) was conducted to determine the PIEVS factorial structure and test Roberts et al. (14) theoretical categorization for the six dimensions retained for the scale. We also evaluated the scale's convergent and divergent validity. Based on the literature described in the introduction, we hypothesized that the beliefs in instrumental effects of violence (measured by the PIEVS) would be positively correlated with adherence to the sport ethics norm and the propensity to use a disempowering climate. On the opposite, we hypothesized that the PIEVS would be negatively correlated with the propensity to use an empowering climate. Finally, we developed and validated a short-form of the PIEVS, based on the original version of 25 items.

#### Procedures and participants

A convenience sample of adult coaches involved in organized sport at the time of the study was recruited. Participants were recruited on a voluntary basis through different strategies, such as emails sent through sport federations and associations as well as targeted ads on social media. The inclusion criteria was to be 18 years old or more and to coach in organized sport at the time of the survey. Coaches from all sports and from all sport levels were invited to participate in the study. Interested participants accessed an anonymous survey through a hyperlink hosted by a secure, online survey software, Qualtrics. Following this step, they electronically agreed to participate via the completion of a consent form before starting the questionnaire. Because IV is a sensitive topic, a list of resources was included in the consent form. Our sample was composed of 690 participants (see Table 2 for sociodemographic characteristics).

**Table 2:** Participants’ sociodemographic characteristics (*N* = 690).

Age (*n* = 682)	
18–25	101 (14.8%)
26–35	114 (16.7%)
36–45	225 (33.0%)
46–55	171 (25.1%)
56–65	54 (7.9%)
65 and older	17 (2.5%)
Ethnic or cultural group (*n* = 682)	
Canadian or Quebecer	610 (89.4%)
American	20 (2.9%)
First Nations, Inuit, Métis, Aboriginal	12 (1.8%)
Latin American (Central and South America)	9 (1.3%)
African American (Caribbean and West Indies)	5 (0.7%)
Sub-Saharan African (Gabon, Senegal, etc.)	3 (0.4%)
North African (Maghreb)/Middle East	3 (0.4%)
Asian (China, Japan, Laos, Philippines, India, etc.)	2 (0.3%)
Western European (France, Spain, etc.)	1 (0.1%)
Eastern European (Hungary, Romania, etc.)	17 (2.5%)
More than one	610 (89.4%)
Sex at birth (*n* = 682)	
Male	455 (66.7%)
Female	227 (32.9%)
Gender identity (*n* = 689)	
Man/Male	453 (65.7%)
Woman/Female	231 (33.5%)
Indigenous or other cultural gender identity (e.g., two-spirit)	3 (0.4%)
Non-binary, gender fluid or something else (e.g., genderqueer)	2 (0.3%)
Type of sport coached (*n* = 659)	
Individual	456 (69.2%)
Team	197 (29.9%)
Both	6 (0.9%)
Coaching experience in years (*n* = 677)	*M* = 12.1, *SD* = 10.4
Level of competition (*n* = 688)	
Local or regional	284 (41.3%)
Provincial	226 (32.8%)
National	118 (17.2%)
International	60 (8.7%)

#### Measures

##### Sociodemographics

Participants provided information regarding the nature of the sport in which they were coaching (team, individual, or both), the level of competition (local/regional, provincial, national, or international), and the years of experience they had in coaching. General sociodemographic characteristics (age, sex, cultural identity, and gender) were also collected.

##### Beliefs regarding instrumental effects of violence

The initial version (25 items) of the Perceived Instrumental Effects of Violence Scale (PIEVS) that we developed in Study 1 was used.

##### Sport ethic norms

To assess convergent validity, we used the Conformity to Sport Ethic Scale [CSES, (34)]. This validated tool measures the participant's degree of adherence to sport ethic norms. The CSES is composed of the “striving for distinction”, the “self-sacrifice”, and the “refusing to accept limits” subscales. For this study, the scale was adapted to fit for coaches. It showed good internal consistency, with Cronbach's coefficient of 0.831.

##### Motivational climate

To assess convergent and divergent validity, we used two subscales of the coach-created Empowering and Disempowering Motivational Climate Questionnaire [EDMCQ-C, (35)], namely Empowering (divergent validity) and Disempowering subscales (convergent validity). This scale measures the motivational climate created by coaches with empowering climates focused on task-involving and autonomy-supportive strategies and disempowering climates focused on ego-involving and controlling strategies (35). In this sample, the Empowering subscale showed good reliability (*α* = 0.830), and the Disempowering had acceptable reliability (*α* = 0.749).

#### Data analysis

An EFA was performed using Mplus version 8.0 (36) to identify the scale's latent dimensions with the objective of obtaining the most parsimonious and conceptually sound factor structure. We used the following criteria to evaluate the best model: items loading above 0.4 and limited cross-loading on more than one factor (37, 38). The overall model fit was evaluated with a combination of fit indices: the chi-square, the *Root Mean Square Error of Approximation* (RMSEA ≤0.05), the *Standardized Root Mean Square Residual* (SRMR ≤0.05), the *Tucker Lewis Index* (TLI ≥0.95), the *Comparative Fit Index* (CFI ≥0.95), and the ratio of chi-square to degrees of freedom (*χ*^2^/df ≤ 3) as fit indices to identify the best solution (30, 39). After determining the best factorial solution, descriptive and correlational analyses were computed with the Statistical Package for the Social Sciences (SPSS 24.0) with a significance level of *p* < .05. The scale's internal consistency was measured with Cronbach alpha (40) and McDonald's omega (41, 42) coefficients. As recommended, we aimed for a score of 0.7 aimed for both coefficients to show adequate reliability (43). For divergent and convergent validity, we measured the correlations between the PIEVS and the three conceptually related scales presented earlier in our measure section, namely the CSES (34), and the EDMCQ-C (35).

In a second step, we developed a short form of the PIEVS to increase its usability in future studies. We conducted an EFA with the 9 items that had obtained the highest factor loadings in the previous stage of analyses. The model fit was evaluated with the same combination of indices, and we measured reliability. We also documented divergent and convergent validity with the previously described scales.

#### Results

##### Factorial structure

We used the Weighted Least Squares Mean and Variance-adjusted (WLSMV) estimator as we dealt with ordered categorical indicators. We compared factorial structures ranging for 1- to 6-factor solutions. The 1-factor solution (11.174), 2-factor solution (1.406), 3-factor solution (1.043), and 4-factor solution (1.028) had Eigenvalues over 1, which is the threshold recommended for continuing the exploration. The screeplot indicated that a 1- or 2- factor solution could be appropriate. Overall, the 1-factor solution appeared highly superior to the others. We continued exploring the 1- to 4-factor solution and saw that they all presented good to excellent fit. The 2- to 4-factor solutions had a few issues of low factor loadings and cross-loadings. We then considered the nature of each factor by evaluating the items that loaded on each to see which solution would be more coherent with the theory on instrumental effects of violence. None of the solutions included factors that corresponded to the six theoretical dimensions identified in Roberts and colleagues' (2020) systematic review. At the end of this process, we decided that the one-factor solution was the most appropriate when considering both psychometric and theoretical relevance. This one-factor model provided an excellent fit to the data, *χ*^2^(275) = 594.38, *p* < .001; RMSEA = .041, 90% CI[.037 to.046]; SRMR = 0.056; CFI = 0.965; TLI = 0.962; *χ*^2^/df = 2.161. Standardized factor loadings of the items are presented in Table 3.

**Table 3:** Factor loadings for the PIEVS.

Item number	Summary of content^a^	Factor loading
1	Yelling or swearing for motivation	.727
2	Ignoring for motivation	.688
3	Asking to reduce social network for performance	.537
4	Mentioning being ashamed for motivation	.769
5	Giving extra workouts after poor performance	.693
6	Being angry after poor performance	.738
7	Shaking or pushing after poor performance	.597
8	Punishing team for one person's mistake	.617
9	Benching after poor performance	.611
10	Accepting yelling as commitment	.697
11	Giving harder training for less committed	.741
12	Ignoring the less committed	.640
13	Bullying as preparation for competition	.736
14	Considering athletes weak or lazy for failure in hard training	.730
15	Imposing pain to increase endurance	.569
16	Restricting food or water to increase endurance	.614
17	Creating sense of fear to obtain obedience	.714
18	Imposing punishments for disrespect	.503
19	Imposing punishments for missed practice	.650
20	Threatening to stop working with an athlete for discipline	.574
21	Removing athletes for weight reasons	.632
22	Maintaining distrust among athletes to promote competition	.770
23	Giving preferential treatment to motivate others	.626
24	Creating conflict among athletes to motivate	.735
25	Openly comparing athletes’ bodies to promote competition	.432

^a^ This Table presents a summary of each item's content. For the full items, please contact the lead researcher.

##### Reliability

The internal consistency of the PIEVS was very good with a Cronbach alpha of 0.891 and a McDonald omega of 0.949. Based on those results and the fact that EFA allow to keep the 25 original items, it was decided to also look for the validation of a short version of the PIEVS (see below—section on short form).

##### Convergent and divergent validity

As expected, the PIEVS and the Empowering subscale were negatively correlated (-.29, *p* < .001), which supported divergent validity. The PIEVS was positively correlated with the Disempowering subscale (.558, *p* < .001) and with the CSES (.653, *p* < .001), which supported convergent validity. The convergent correlations were strong but not near perfect, which was consistent with similar yet distinct concepts being measured.

##### Short-Form

We performed the second EFA also using the WLSMV. We compared factorial structures ranging for 1- to 4-factor solutions given the results from the first EFA. When looking at the Eigenvalues, we saw that only the 1-factor solution (5.340), was over 1, which is the threshold recommended for continuing the exploration. The screeplot clearly indicated that a 1-factor solution was the most appropriate. This one-factor model provided an excellent fit to the data, *χ*^2^(27) = 66.946, *p* < .001; RMSEA = .046, 90% CI [.032–.060]; SRMR = 0.041; CFI = 0.986; TLI = 0.982; *χ*^2^/df = 2.479. Standardized factor loadings of the items are presented in Table 4. The internal consistency of the PIEVS short form was very good with a Cronbach alpha of 0.82 and a McDonald omega of 0.917. Concerning convergent and divergent validity, the PIEMS short form and the Empowering subscale were negatively correlated (−0.27, *p* < .01), which supported divergent validity. The PIEVS short form was positively correlated with the Disempowering subscale (0.476, *p* < .01) and with the CSES (0.556, *p* < .01), which supported convergent validity.

**Table 4:** Factor loadings for the PIEVS short-form.

Item number	Summary of content^a^	Factor loading
1	Yelling or swearing for motivation	.761
4	Mentioning being ashamed for motivation	.779
6	Being angry after poor performance	.729
11	Giving harder training for less committed	.713
13	Bullying in training to prepare for competition	.766
14	Considering athletes weak or lazy for failure in hard training	.674
17	Creating sense of fear to obtain obedience	.709
22	Maintaining distrust among athletes to promote competition	.782
24	Creating conflict among athletes to motivate	.769

^a^ This Table presents a summary of each item's content. For the full items, please contact the lead researcher.

## Discussion

The present study aimed to develop and validate a scale that assesses sport coaches' beliefs regarding instrumental effects of violence, namely the *Perceived Instrumental Effects of Violence Scale* (PIEVS). To our knowledge, the PIEVS is the first measurement tool for this concept. Based on the two studies reported in this paper, we obtained a one-factor, 25-item scale with a very good internal consistency and an excellent fit to the data. We also obtained a 1-factor, 9-item short scale with satisfying psychometric characteristics. These one-factor models differ from the theoretical 6-factor model based on the proposed dimensions from Roberts et al. (14) that we chose to test (drive performance, deter failure, test resilience, and commitment, develop toughness, assure interpersonal control, and promote internal competition). This could be explained by the fact that these dimensions are all related to the same global goal: to assure compliance of athletes with the demands, expectations, and goals of experts in positions of authority. As stated by Roberts et al. (14), “abuse is construed as an effective and acceptable way to discipline value-inconsistent behaviour” (p. 23). This means that the sport culture is dictating a set of values (“dominant values”—as described by Roberts and her colleagues) that coaches, among others, endorse. These dominant values (high-performance values, traditional masculine values, and expertise values) could explain the perceived need to ensure that athletes' behaviors fit this normative framework. It also logically explains that we found convergent validity with the *Conformity to the Sport Ethic Scale* (34). Indeed, a previous study showed an association between CSES scores and coaches' IV behaviors, as reported by athletes (23). We could then hypothesize that perceived instrumental effects of violence could be a mediating variable between dominant values in sport and IV from coaches. This hypothesis remains to be tested.

Coaches' beliefs about the perceived instrumental effects of violence could also be related to the motivational climate they strive to implement among their teams. An empowering climate combines task-involving, autonomy-supportive, and socially-supportive strategies (35). Meanwhile, a disempowering climate focuses on ego-involving and controlling strategies (35). As described previously, the PIEVS' factorial structure suggests that the underlying “goal” of violence is to help coaches guide athletes toward value-consistent behaviors. It is therefore not surprising that we found convergent validity with the Disempowering subscale and divergent validity with the Empowering subscale of the *Motivational Climate Questionnaire* (35). As stated in the introduction, Ohlert and colleagues (24) have recently observed that a disempowering climate constituted a risk factor for IV. On the contrary, an empowering climate could serve as a protective factor. More research is needed to increase our understanding of the relation between these factors and ultimately develop more efficient prevention strategies. As such, the PIEVS could be used in future studies to evaluate if programs promoting the use of empowering motivational climates also influence the coaches' beliefs concerning the perceived instrumental effects of violence.

The development and validation of the PIEVS open many avenues for future research to refine our understanding of the various factors that can explain IV in sport. Indeed, the tool could be used to test associations between beliefs regarding instrumental effects of violence and actual coach IV behaviors (observed or self-reported). We could then pursue further and document if coaches' beliefs about the instrumental effects of violence are related to the dominant values identified in sport by Roberts et al. (14). This could lead to testing our hypothesis on the possible mediation role of such beliefs between dominant values and IV in sport. If the results of these studies support the relationship between beliefs regarding instrumental effects of violence and actual IV behaviors, the PIEVS could then be used to evaluate changes in attitudes and beliefs of coaches in the context of an intervention aimed at changing behaviors regarding IV in sport.

Although our study focused on coaches, given their central role, it is essential to underline that they are only one part of the sport ecosystem. As reported in previous studies, athletes, parents, and administrators are all concerned by the dominant values related to IV in sport [(14, 23), p. 16]. This means that other actors in sport could also endorse the perceived instrumental effects of violence. In turn, the situation creates a reinforcing loop that contributes to coaches maintaining those attitudes, beliefs, and behaviors. Adaptations of the PIEVS to these actors could be interesting and will need to be tested for validity.

### Limitations

While we believe that the PIEVS represents a promising measurement tool to further improve both research and intervention regarding IV in sport, limitations should be noted. The tool was developed to measure coach beliefs only, and does not allow for the measurement of other sport actors' beliefs. This research was conducted with a convenience sample of coaches from Québec, Canada. The PIEVS should be tested in other samples in other cultures and regions to confirm the obtained factorial structure. To do so, some cultural adaptations and translations will be needed. There could be differences in beliefs concerning perceived instrumental effects of violence based on gender, sports, years of experience, coach education levels, competition levels, and cultures. Future research with the PIEVS should document such differences and use the findings to refine the scale if appropriate.

## Conclusion

In conclusion, the process of developing the PIEVS led us to reflect on the explanation of coaches' IV behaviors. As explained previously, more sport-specific tools to measure diverse potential contributing factors, such as sport-specific social norms and other organizational factors are needed. The PIEVS could serve as a tool to measure progress, but it is not the only measurement tool that we need to fully understand the whole picture and explain more fully why coaches use IV toward athletes in their practice. As long as we do not have a clear model to explain coaches' IV behaviors, it will be difficult to fully understand how to prevent this phenomenon in sport. The PIEVS is one step toward this end.

## Data Availability

The raw data supporting the conclusions of this article will be made available by the authors, without undue reservation.
